# First echinoderm alpha-amylase from a tropical sea cucumber (*Holothuria leucospilota*): Molecular cloning, tissue distribution, cellular localization and functional production in a heterogenous *E.coli* system with codon optimization

**DOI:** 10.1371/journal.pone.0239044

**Published:** 2020-09-15

**Authors:** Xiaofen Wu, Yao Ruan, Ting Chen, Zonghe Yu, Da Huo, Xiaomin Li, Feifei Wu, Xiao Jiang, Chunhua Ren

**Affiliations:** 1 CAS Key Laboratory of Tropical Marine Bio-resources and Ecology (LMB), Guangdong Provincial Key Laboratory of Applied Marine Biology (LAMB), South China Sea Institute of Oceanology, Chinese Academy of Sciences, Guangzhou, PR China; 2 University of Chinese Academy of Sciences, Beijing, PR China; 3 Southern Marine Science and Engineering Guangdong Laboratory (Guangzhou), Guangzhou, PR China; 4 Institution of South China Sea Ecology and Environmental Engineering, Chinese Academy of Sciences, ISEE, CAS, Guangzhou, PR China; 5 College of Marine Sciences, South China Agricultural University, Guangzhou, PR China; Laboratoire Arago, FRANCE

## Abstract

*Holothuria leucospilota* (*Echinodermata*: *Holothuroidea*) is a widespread tropical sea cucumber with strong value for the ecological restoration of coral reefs. Therefore, some studies regarding the artificial breeding and cultivation of *H*. *leucospilota* have been undertaken recently. However, the biological functions of the digestive system of this species have not been elucidated. In this study, a cDNA coding for α-amylase, an indicator of digestive maturity in animals, was identified from *H*. *leucospilota* and designated *Hl-Amy*. The full-length cDNA of the *Hl-Amy* gene, which is 1734 bp in length with an open reading frame (ORF) of 1578 bp, encodes a 525 amino acid (a.a.) protein with a deduced molecular weight of 59.34 kDa. According to the CaZy database annotation, Hl-Amy belongs to the class of GH-H with the official nomenclature of α-amylase (EC 3.2.1.1) or 4-α-D-glucan glucanohydrolase. The Hl-Amy protein contains a signal peptide at the N-terminal followed by a functional amylase domain, which includes the catalytic activity site. The mRNA expression of *Hl-Amy* was abundantly exhibited in the intestine, followed by the transverse vessel with a low level, but was hardly detected in other selected tissues. During embryonic and larval development, *Hl-Amy* was constitutively expressed in all stages, and the highest expression level was observed in the blastula. By *in situ* hybridization (*IS*H), positive *Hl-Amy* signals were observed in different parts of the three different intestinal segments (foregut, midgut and hindgut). The Hl-Amy recombinant protein was generated in an *E*. *coli* system with codon optimization, which is necessary for Hl-Amy successfully expressed in this heterogenous system. The Hl-Amy recombinant protein was purified by immobilized metal ion affinity chromatography (IMAC), and its activity of starch hydrolysis was further detected. The optimal temperatures and pH for Hl-Amy recombinant protein were 55°C and 6.0, respectively, with an activity of 62.2 U/mg. In summary, this current study has filled a knowledge gap on the biological function and expression profiles of an essential digestive enzyme in sea cucumber, which may encourage future investigation toward rationalized diets for *H*. *leucospilota* in artificial cultivation, and optimized heterogenous prokaryotic systems for producing recombinant enzymes of marine origins.

## Introduction

Amylase is a general term for a class of enzymes that catalyze the hydrolysis of glycosides in starch, glycogen and dextrin. Naturally, amylase is widespread in almost all living organisms. According to the different ways in which the amylases act on starch, they are classically divided into three majority categories: α-amylase (EC 3.2.1.1), β-amylase (EC 3.2.1.2) and γ-amylase (EC 3.2.1.3). Among these categories, α-amylase (EC 3.2.1.1, 1,4-α-D-glucan-glucanohydrolase) is the most common type and is an extracellular enzyme that hydrolyzes starch and glycogen molecules [1]. This enzyme breaks down the α-1,4-glycosidic bonds in long-chain saccharides into glucose, maltose, maltotriose and α-limit dextrin [2, 3]. Amylase plays an important role in carbohydrate metabolism *in vivo*, and the alpha-amylase genes therefore can be found in various organisms, from bacteria [4], fungi [5], plants [6] to animals [7]. In vertebrates, the α-amylase gene was first cloned from mice in 1982 [7]. The α-amylase gene was cloned from *Drosophila melanogaster* in 1985, representing the first invertebrate α-amylase gene to be characterized [8], followed by other cases for invertebrate species that reported in *Crassostrea gigas* [9], *Pinctada fucata* [10] and *Ciona intestinalis* [11]. Echinoderms are a variety of invertebrate marine animals belonging to the phylum *Echinodermata*, which is the second-largest group of deuterostomes, after the chordates. However, the identification of amylase and its biological and biochemical properties in echinoderms have not been characterized so far.

Sea cucumbers belong to the class *Holothuroidea* of phylum *Echinodermata*. In the marine ecosystem, sea cucumbers serve as scavengers based on their contributions to recycling nutrients and breaking down detritus and other organic matter [12]. *Holothuria leucospilota* is a tropical sea cucumber species that is naturally distributed in the Indo-Pacific region [13]. *H*. *leucospilota* can live in either the nutrient-rich nearshore area or the nutrient-poor coral reef area [14, 15]. Therefore, artificial breeding and culture for *H*. *leucospilota* have been developed recently for future applications in the restoration of the ecological system of coral reefs [16]. However, little is known regarding the basic digestive knowledge and the mechanism of carbohydrate metabolism of this species.

Studies regarding the digestive pattern of *H*. *leucospilota* remain limited, except for a previous report for the identification of trehalase in this species [17]. To further understand the biological basics of the digestive system in sea cucumber, an α-amylase, which is considered an indicator of digestive maturity in most animals [18], was identified from *H*. *leucospilota* in this study and designated as *Hl-Amy*. We described the structural and phylogenetic characteristics of Hl-Amy, detected its expression profiles among different tissues, and embryonic and larval stages and localized its expressed cells in the intestine. We further expressed and purified the recombinant protein of Hl-Amy in a prokaryotic *E*. *coli* system with codon optimization. The activities of Hl-Amy recombinant protein were demonstrated on starch hydrolysis with optimization of its functional temperatures and pH. This study aims to elucidate the structural, phylogenetic, transcriptional, developmental and functional characteristics of α-amylase in echinoderms, and to improve a methodological framework for utilizing heterogenous prokaryotic systems to generate such enzymes from marine animals.

## Materials and methods

### Ethics statement

In this study, the sea cucumber were collected from commercial catches. Therefore, approval from any ethics committee or institutional review board was not necessary. No ethics approval was required for the collection or experimentation of the animal used. Sea cucumbers belong to lower invertebrate animals, which does not have a complete nervous system. The animal experiments were conducted in accordance with the guidelines and approval of the Ethics Committees of South China Sea Institute of Oceanology, Chinese Academy of Sciences.

### Animals and collection of tissue, embryonic and larval samples

For molecular cloning and tissue distribution, healthy sea cucumbers (*H*. *leucospilota*) with weights of 200–280 g were obtained from Daya Bay in Shenzhen, Guangdong Province, China, and kept in an aquarium with filtered seawater (30‰ salinity) and accompanying continuous aeration at 30°C for a week before experiments were performed. The sea cucumbers were dissected on ice, the tissues were collected and RNA was extracted immediately to avoid degradation. To harvest the coelomocytes, the coelomic fluids were collected through a 150 mesh to remove large tissue debris and centrifuged immediately at 8000×g for 10 min at 4°C. Then, 1 ml TRIzol reagent (Invitrogen, USA) was used to dissolve the obtained coelomocytes. Embryonic and different developmental periods of larval samples were collected as described previously [17] from the Jinyang aquaculture base in Maoming city, Guangdong Province, China. Every stage’s sample contain several individuals with weights of 50–100 mg, and we collect three sample in each development stage. Then, these samples were frozen in RNAlater^™^ Soln (Invitrogen, Carlsbad, CA, USA), and all samples were stored at −80°C for further studies.

### Molecular cloning of Hl-Amy full-length cDNA

Sea cucumber intestinal total RNA extraction and reverse transcription were performed as previous description [17]. The gene-specific primers (S1 Table) used in cDNA cloning, including the middle segments verification, 3’- and 5’-RACE were designed based on a gene of 1494 bp from an unpublished *H*. *leucospilota* transcriptome which was previously constructed in our group (S1 Data). The amplification conditions for middle segments verification and RACE were 35 cycles of 94°C for 30 s, 56°C for 30 s and 72°C for 2 min. The pMD-18-T vector (TaKaRa, Japan) and DH5α (JM109) competent cells were used for subcloning the amplicons. In this case, three positive clones of each amplicon were sequenced.

### Bioinformatics analysis

Official nomenclature was determined according to CaZy database based on Hl-Amy amino acid sequence (http://bcb.unl.edu/dbCAN2/). Structural domains of *Hl-Amy* were predicted by using the SMART (http://smart.embl-heidelberg.de/), ScanProsite (http://prosite.expasy.org/) and BLAST (https://blast.ncbi.nlm.nih.gov) programs. The 3D models were deduced with Swiss modeling software provided by the SWISS-MODEL server (https://swissmodel.expasy.org/) and visualized by VDM 1.9.2. Alignment for a.a. sequences among various species was performed with the ClustalX program and demonstrated using the GeneDoc program. A phylogenetic tree was constructed based on a.a. difference (p-distance) obtained with the neighbor-joining method (pairwise deletion) with 1000 bootstrap replicates using MEGA 7.0.

### Tissue distribution and ontogeny of Hl-Amy mRNA expression

The tissue distribution of *Hl-Amy* mRNA was quantitatively detected in three individuals. The selected tissues included the intestine, transverse vessel, esophagus, cuvierian tubules, respiratory trees, body wall, muscle, polian vesicle, coelomocytes, rete mirabile, ovary and testis. The embryos and larvae were sampled in nine developmental stages according to their morphologies as observed using an optical microscope, including blastula, early gastrula, late gastrula, early auricularia, mid auricularia, late auricularia, doliolaria, pentactula and juvenile.

Total RNA was extracted with TRIzol reagent and reverse transcribed into first strand cDNA using the PrimeScript^™^ RT reagent Kit with gDNA Eraser (Perfect Real Time, TaKaRa). Specific primers *qHl-Amy-F* and *qHl-Amy-R* (S1 Table) were designed based on the obtained *Hl-Amy* cDNA sequence. Real-time PCR was carried out using a Thermal cycler dice real time system III (TaKaRa) in a 20-μl reaction, under a two-step method in the condition of 40 cycles of 95°C for 5 s and 60°C for 30 s. In this case, the housekeeping gene *HLβ-actin* was used as an internal control referring to our previous studies [19]. The levels of *Hl-Amy* were analyzed using the 2^-ΔΔCt^ method, and the data were routinely normalized as a ratio of *HLβ-actin* mRNA detected in the same sample.

### *In situ* hybridization for Hl-Amy in the intestine of sea cucumber

The *H*. *leucospilota* intestine was collected by being divided it into three parts: the foregut, midgut and hindgut. For *in situ* hybridization (*IS*H), the obtained samples were proceeded as previous description [17]. The digoxigenin (DIG)-dUTP-labeled DNA probe against *Hl-Amy* was generated by PCR DIG Probe Synthesis Kit (Sigma-Aldrich, Germany) with primer p-Amy-F and p-Amy-R (S1 Table). The *IS*H signal was detected by a diaminobenzidine (DAB) method with incubation of horseradish peroxidase (HRP)-conjugated anti-DIG antibody, and the nucleus were restained with hematoxylin. A Case Viewer system was used for the figure observation. An absence of *Hl-Amy* DNA probe was performed as negative control and the haematoxylin and eosin (H & E) stain was the parallel experiment for histology observation.

### Expression and purification of recombination protein of Hl-Amy

The codons for the first 20 amino acids after the signal peptide in the ORF of *Hl-Amy* were optimized without changing the original amino acid sequence (S2 Data). Specifically, using the primer designed for codon optimization in S1 Table to perform three round PCR with Amy-CO-F1(for removal of the original 20 first aa after signal peptide in the ORF), Amy-CO-F2 (including the optimized codon) and Amy-CO-F3 (including the *Sca*I site) as forward primer respectively and Amy-Xho1-R (including *Xho*I site.) as reverse primer. Using plasmid containing the purpose gene as templates in the first round PCR. Diluting the product from last round PCR by 100 times as the template in the next round with the PCR procedure as 35 cycles of 94°C for 30s, 60°C (56°C in the first round) for 30 s and 72°C for 2 min (1.5 min in the first round). Then subcloned into the vector pET28a which linearized by enzyme *Sca*I and *Xho*I with the ClonExpress II One Step Cloning Kit (Vazyme, Nanjing). The transformation of Hl-Amy and pET28a recombinant expression vector, condition of culture and induced expression, and recombinant protein purification were performed as previously described [20]. Protein concentration was determined according to Lowry’s method using bovine serum albumin as a standard.

### Activity assay of Hl-Amy

The activity of the purified Hl-Amy protein was assayed using the α-Amylase Assay Kit (Solarbio, China), where the standard curve of D-glucose was determined by following its protocol. The Hl-Amy activity was determined using soluble starch (final concentration of 0.5%) as a substrate. The 1.0% soluble starch volume of 75 μL was added with equivalent amylases at a concentration of 1 mg/mL that was dissolved in 10 mM Tris-maleate buffer containing 300 mM NaCl, and the mixture was incubated at 40°C for another 10 min. The enzymatic reaction was then stopped by the addition of 150 μL of 4 mM 3,5-dinitrosalicylic acid. After the color development was promoted by heating at 90°C for 10 min, the reducing sugar reacted with 3,5-dinitrosalicylic acid and formed a red color compound. The reaction products were cooled to room temperature, and the absorbance at 562 nm (A_562_) was measured with Multiskan Ascent (Thermo Scientific, USA). One unit of enzyme activity was defined as the amount of amylase that could cause an increase in A_562_ of 1 unit within 10 min at 40°C. Specific activity was expressed as units of enzymatic activity per milligram of protein.

The optimal temperature assay was carried out by adding 75 μL of the recombinant Hl-Amy protein into an equivalent reaction mixture (0.5% soluble starch in 10 mM Tris-maleate buffer, pH 7.0) and then incubated at various temperatures (20, 25, 30, 35, 40, 45, 50, 55, 60, 65, 70, 75, 80, 85, and 90°C) for 10 min. The optimal pH assay was carried out by adding 75 μL of the recombinant Hl-Amy protein into an equivalent reaction mixture at different pH values (3.5, 4.0, 4.5, 5.0, 5.5, 6.0, 6.5, 7.0, 7.5, 8.0, 8.5, 9.0, 9.5 and 10.0, which were adjusted by 0.2 M citrate buffer, 0.2 M phosphate buffer and 0.2 M bicarbonate buffer) and incubated at 40°C for 10 min. The reaction was stopped by the addition of 150 μL of 4 mM 3,5-dinitrosalicylic acid. After 10 min of heating at 90°C, the reaction mixtures were cooled to room temperature, and then the values of A_562_ were measured with Multiskan Ascent. The relative enzyme activities at different temperatures and pH were calculated, with the highest enzyme activity being 100% [21].

## Results

### Molecular cloning and sequence analysis of Hl-Amy

**Fig 1:**
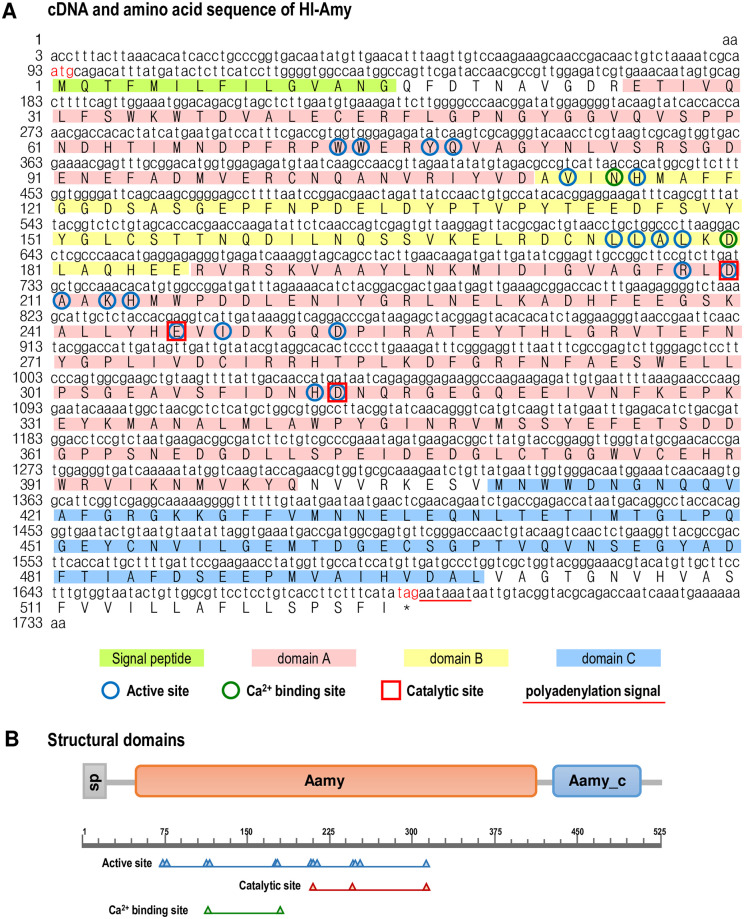
Full-length cDNA sequence and structural analysis of Hl-Amy. **A**: Full-length cDNA sequence and deduced amino acid sequence of *Hl-Amy*. The translational start codon (atg) and stop codon (taa) are shown in red, and the polyadenylation signal (aataaa) in the 3’-UTR is underlined. The signal peptide, domain A, domain B and domain C are shown in the boxes with different colors, and the active site, Ca^2+^ binding site and catalytic site are indicated with different symbols. **B**: Structural domain of Hl-Amy predicted using the SMART, ScanProsite and BLAST programs. The signal peptide (SP), α-amylase domain (Aamy) and α-amylase C-terminal domain (Aamy_C) are boxed, and the active site, Ca^2+^ binding site and catalytic site are shown.

Using 3’-/5’-RACE approaches, the full-length cDNA sequence of Hl-Amy was obtained with GenBank No. MN592681. The *Hl-Amy* cDNA is 1734 bp in length, containing a 5’-untranslated region (UTR) of 92 bp, a 3’-UTR of 36 bp and an open reading frame (ORF) of 1578 bp that encodes a 525 amino acid (a.a.) protein with a deduced molecular weight of 59.34 kDa and a predicted isoelectric point of 4.54 (Fig 1A). Hl-Amy belongs to the class of GH-H (glycoside hydrolases) with its accepted name is α-amylase (EC 3.2.1.1), systematic name is 4-α-D-glucan glucanohydrolase and reacts on endohydrolysis of (1→4)-α-D-glucosidic linkages in polysaccharides containing three or more (1→4)-α-linked D-glucose units. The Hl-Amy protein contains a signal peptide (Met^1^–Gly^15^) at the N-terminal, followed by a TIM barrel containing the active site residues and chloride ion-binding site (Glu^26^–Asp^122^ and Arg^187^–Gln^401^, domain A), a long loop region inserted between the third β-sheet and the α-helix of domain A that contains calcium-binding site (Ala^123^–Glu^186^, domain B), and a C-terminal beta-sheet domain (Ala^410^–Glu^499^, domain C) (Fig 1A). By structural prediction, the SMART Aamy domain and Aamy_c domain, and the active site, calcium-binding site and catalytic site were further indicated (Fig 1B).

### Phylogenetic, homology and structural analysis

**Fig 2:**
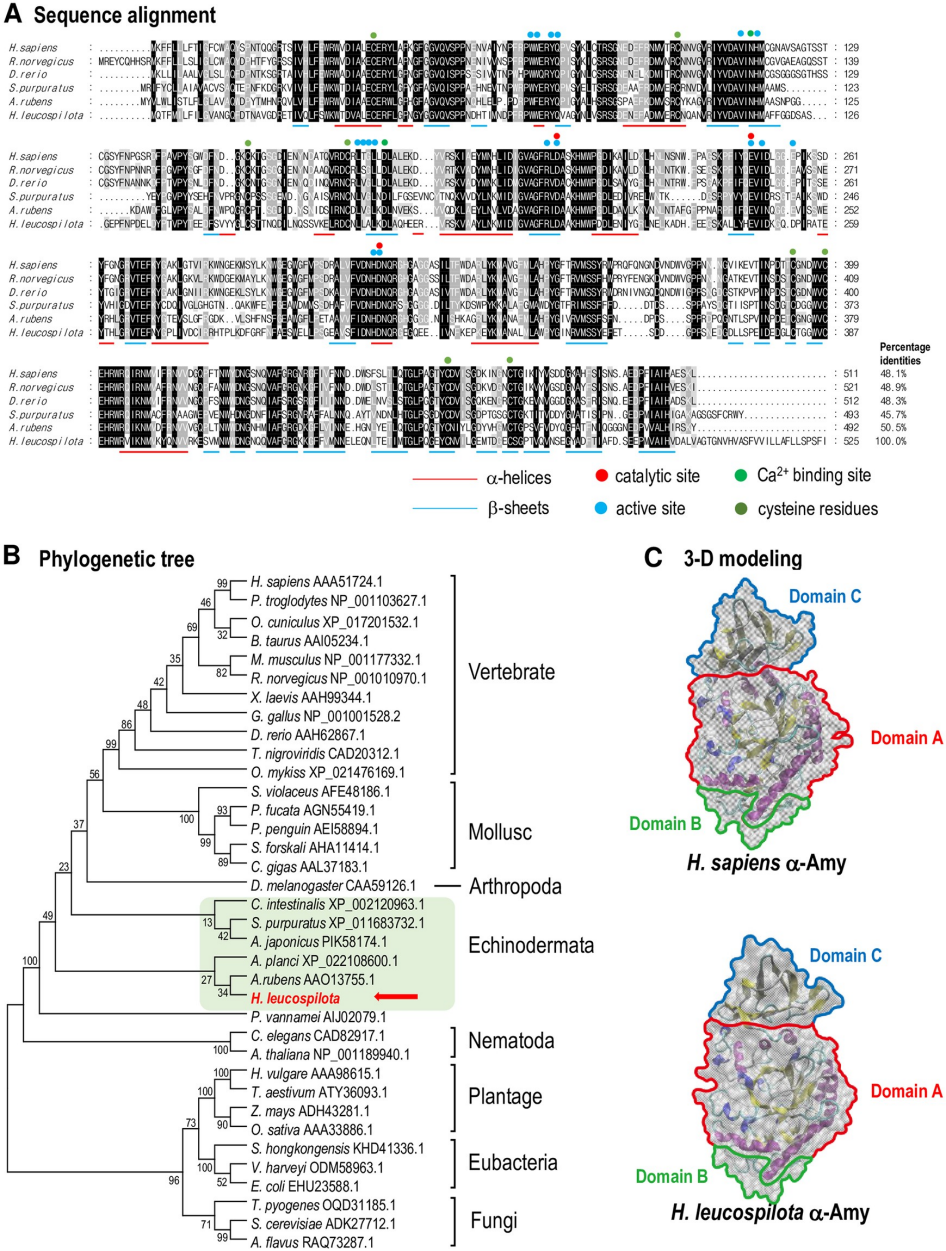
Amino acid sequence alignment, phylogenetic and 3D structure analysis of Hl-Amy. **A**: Amino acid sequence alignment of Hl-Amy and amylase in other deuterostome animal species. The conserved amino acid residues are boxed in dark gray and similar amino acid residues are labeled in light gray. The α-helices, β-sheets are underlined the conserved cysteine residues are indicated, and the active site, Ca^2+^ binding site and catalytic site are shown. **B**: Phylogenetic analysis of amylase in various species using the Neighbor-Joining method with a bootstrap value of 1000. The amylases of echinoderms are boxed, and our newly identified *Hl-Amy* is shown with a red arrow. **C**: Comparison of the 3D structures models for human (*H*. *sapiens*) and sea cucumber (*H*. *leucospilota*) amylases. The structural domain A, B and C are indicated.

Multiple alignment revealed that the Hl-Amy a.a. sequence shared 45.7%–50.5% identities with other known amylases (Fig 2A). In this case, the amylases from different animal species were highly conserved in the active, calcium-binding and catalytic sites for catalytic function and the Cys residues for disulfide bond formation, and relatively similar in the regions of α-helices and β-sheets, but varied in other regions at the primary sequence level. A phylogenetic tree was constructed by a neighbor-joining (NJ) method with amylase a.a. sequences from plants, bacteria, fungi and animals, which include chordates, echinoderms, and nematodes (Fig 2B). The newly identified Hl-Amy, with the shortest evolutionary distance from *Asterias rubens* amylase, was further clustered with vase tunicate (*Ciona intestinalis*), sea urchin (*Strongylocentrotus purpuratus*) and Crown-of-thorns starfish (*Acanthaster planci*) amylases (Fig 2B). The three-dimensional (3D) models were built up with the a.a. sequences of human (*Homo sapiens*) and sea cucumber amylases (Fig 2C). In addition, the 3D structure status of this enzyme is (β/α) 8 with Asp and Glu as its catalytic base and catalytic proton donor respectively base on the CaZy database prediction. Although the α-amylases from human (*H*. *sapiens*) and *H*. *leucospilota* were divergent in primary sequences, their 3-D protein structures shown highly comparable, based on the conservation within their sequences for formation of disulfide bond, α-helix and β-sheet.

### Expression pattern of the Hl-Amy transcript in different tissues

**Fig 3:**
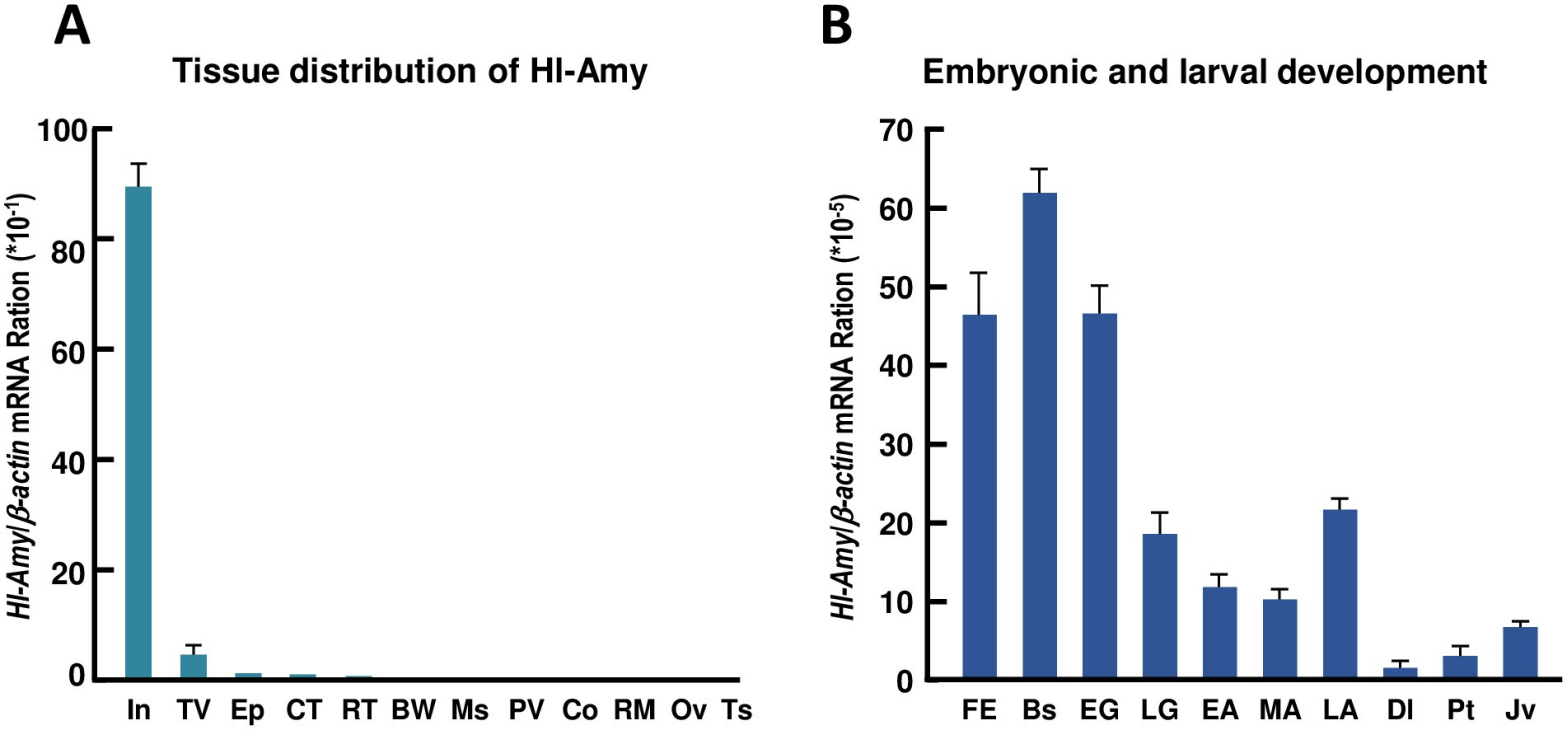
Tissue distribution and embryonic and larval developmental expression of Hl-Amy mRNA. **A**: Expression profiles of *Hl-Amy* mRNA in different tissues of *H*. *leucospilota*, including intestine (In), transverse vessel (TV), esophagus (Ep), cuvierian tubules (CT), respiratory trees (RT), body wall (BW), muscle (Ms), polian vesicle (PV), coelomocytes (Co), rete mirabile (RM), ovary (Ov) and testis (Ts). **B**: Expression profiles of *Hl-Amy* mRNA in embryonic and larval development of *H*. *leucospilota*, including fertilized egg (FE), blastula (Bs), early gastrula (EG), late gastrula (LG), early auricularia (EA), mid auricularia (MA), late auricularia (LA), doliolaria (Dl), pentactula (Pt) and juvenile (Jv). The data presented are expressed as the mean± S.E with three biological replicates.

The mRNA expression of *Hl-Amy* in the selected tissues of *H*. *leucospilota* was analyzed by quantitative real-time PCR. As shown in Fig 3A, *Hl-Amy* was expressed in the intestine with the highest expression level, followed by the transverse vessel, esophagus, Cuvierian tubules, but could hardly be detected in the respiratory trees, body wall, muscle, polian vesicle, coelomocytes, rete mirabile, ovary and testis.

### Expression pattern of Hl-Amy transcript during embryonic and larval development

The expression of *Hl-Amy* mRNA during the embryonic and larval development of *H*. *leucospilota* was determined by quantitative real-time PCR. As shown in Fig 3B, *Hl-Amy* mRNA was constitutively expressed in all embryonic and larval developmental stages that we detected, and the highest expression level was observed at the blastula stage. After that stage, the expression level of *Hl-Amy* mRNA decreased continuously, reached its bottom at the mid-auricularia stage, increased again at the late auricularia stage, reached its bottom again at the doliolaria stage, and then gradually increased. When the sea cucumber developed to the stages of pentactula and juvenile, the mRNA levels of *Hl-Amy* reached a higher level.

### Localization of Hl-Amy positive cells in the sea cucumber intestine

**Fig 4:**
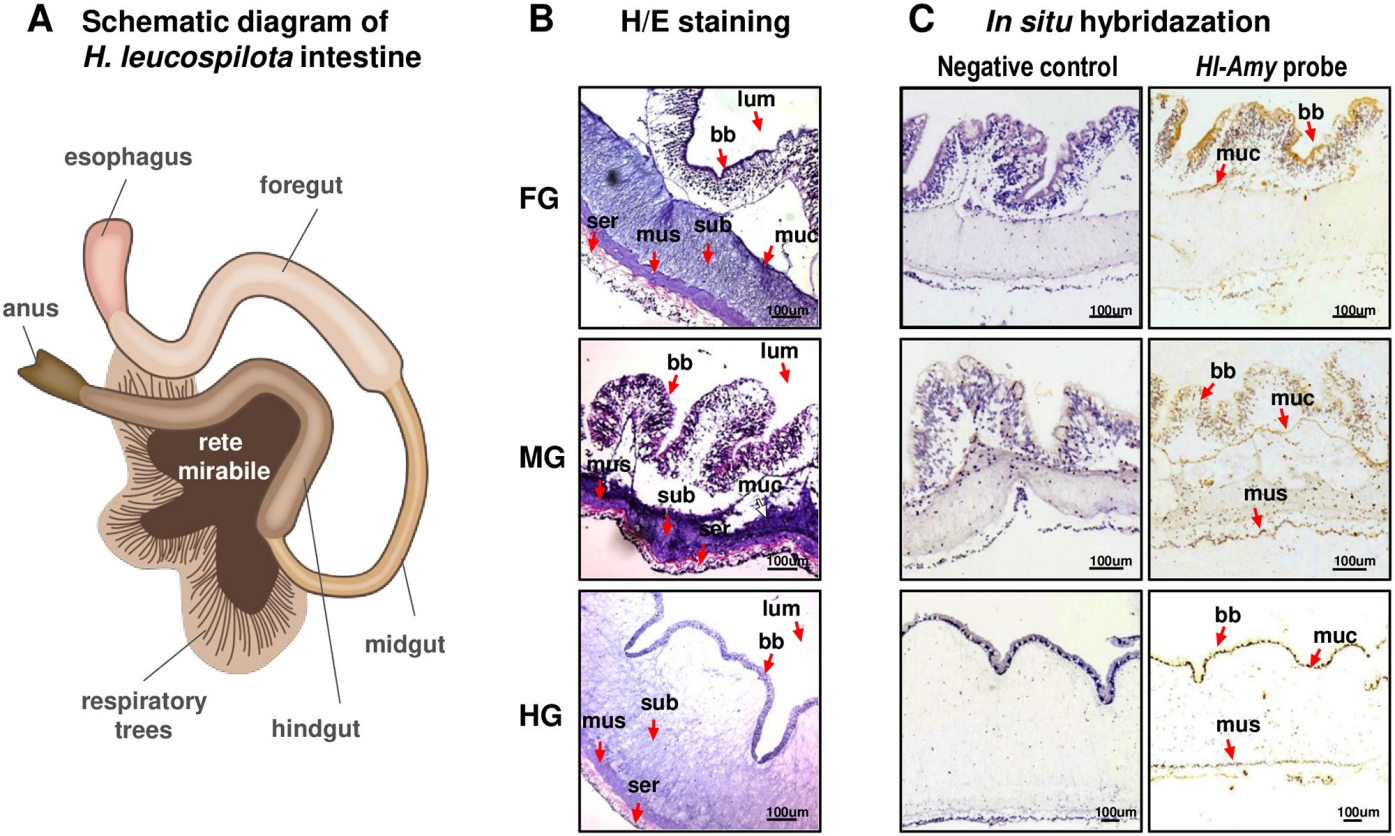
Localization of Hl-Amy positive cells in the sea cucumber intestine. Localization of *Hl-Amy* positive cells were detected in the *H*. *leucospilota* intestine. The intestine is divided into the foregut (FG), midgut (MG) and hindgut (HG). H/E stain is the section stained with hematoxylin and eosin. Negative control of hybridization was performed without a DIG-labeled DNA template. The substructures of the *H*. *leucospilota* intestine included submucosa (sub), serosa (ser), muscle (mus), mucosa (muc), lumen (lum) and brush borders (bb).

In our study, the intestine of *H*. *leucospilota* was divided into three parts, namely, the foregut, midgut and hindgut (Fig 4A), based on their morphologies, for detecting the localization of *Hl-Amy* mRNA-expressed cells using the *in situ* hybridization (*IS*H) approach. As a result, the morphologies of these three intestine segments are different. The foregut and the midgut has a similar structure in which the brush borders are rich, while in the hindgut, the submucosa is considerably thicker than those in the foregut and hindgut, with fewer brush borders (Fig 4B). The *Hl-Amy* mRNA-positive cells were found to localize in these selected parts of the intestine, with the signal being distributed in different parts. Specifically, the *Hl-Amy* mRNA-positive cells were concentrated on the brush borders of the foregut, dispersed on the brush borders, mucosa and muscle of the midgut and strongly and uniquely appeared on the brush borders of the hindgut (Fig 4C). For the negative control slice, no signal was observed in any part of the intestine (Fig 4C).

### Expression and purification of recombination of Hl-Amy protein

**Fig 5:**
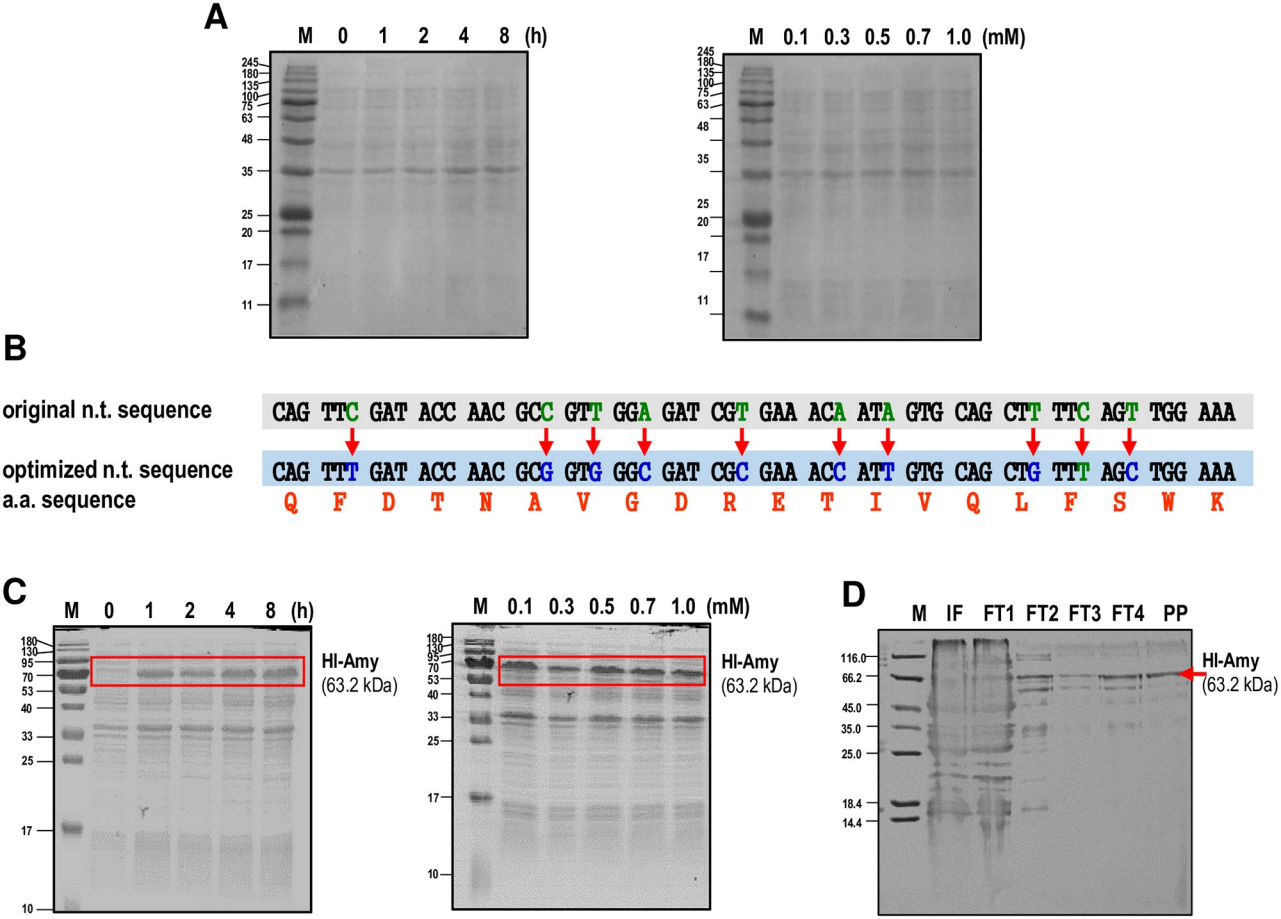
Expression and purification of recombination of Hl-Amy protein. **A**: Codon optimization of *Hl-Amy* mRNA. Original n. t. sequence are shown in green, while optimized n. t. sequence are shown in blue with the optimized codon were highlighted and without changing the A.A. sequence (the red letter). **B**: Time course of IPTG induction for recombinant Hl-Amy protein expression in BL21 *E*. *coli* with its original nucleotide sequence. **C**: Time course of IPTG induction for recombinant Hl-Amy protein expression in BL21 *E*. *coli* with its optimal nucleotide sequence. The cell lysates were collected from 0, 2, 4, 6, and 8 h after 1 mM IPTG induction or collected from 4 h after 0.1, 0.3, 0.5, 0.7, and 1 mM IPTG induction. **D**: Purification of Hl-Amy recombinant protein cell lysate with 0.5 mM IPTG induction. M: Protein marker, IF: insoluble fraction of the cell lysate, FT-1: flow-through sample after washing with binding buffer, FT-2: flow-through sample after 20 mM imidazole elution; FT-3, FT-4: flow-through sample after 50 mM imidazole elution; PP: Purified protein after 500 mM imidazole elution.

For further characterization of the biological activities of Hl-Amy in *H*. *leucospilota*, recombinant Hl-Amy protein was expressed as a C-terminal His-tagged fusion protein in *E*. *coli* and purified by immobilized metal-ion affinity chromatography (IMAC). However, the *E*. *coli* system was not successfully expressed the Hl-Amy protein with its natural nucleotide sequence (Fig 5A&5B). After codon optimization to adapt the codon usage bias of prokaryotic *E*. *coli*, SDS-PAGE analysis showed that the recombinant Hl-Amy protein (63.2 kDa) was successfully expressed, in the conditions of either 1.0 Mm IPTG induction for different hours or different concentrations of IPTG induction for 8 h (Fig 5B&5C), and high-purity protein products with a concentration of 1 mg/mL were finally obtained after IMAC purification and PD-10 desalting (Fig 5D).

### Characterization of the activity and optimal temperature and pH for Hl-Amy

**Fig 6:**
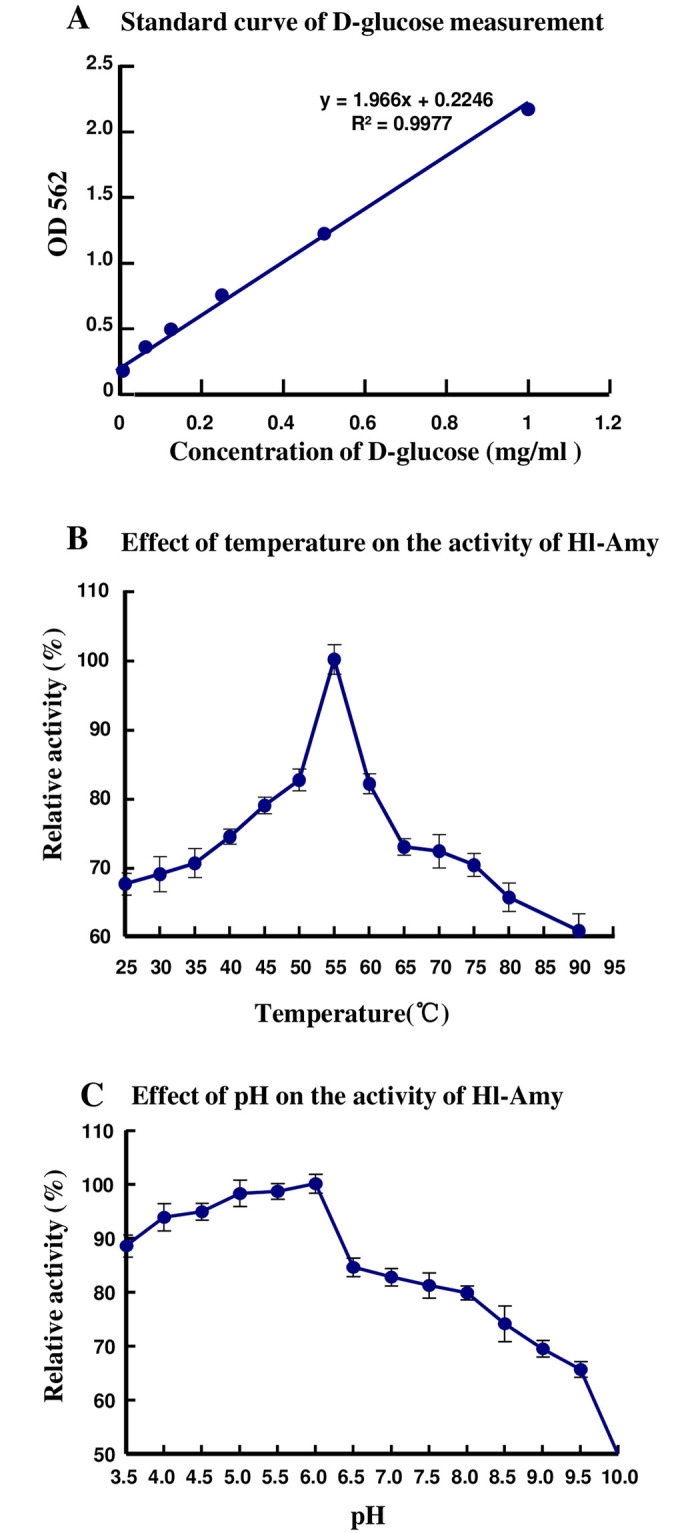
Activity and characterization of Hl-Amy recombinant protein. **A**: Standard curve of D-glucose measurement. The X axis represents the concentration of D-glucose (mg/mL) and the Y axis shows the absorbance of amylase Hl-Amy at 562 nm (A_562_). **B**: Effect of temperature on the activity of Hl-Amy. The X axis represents the value of temperature (°C) and the Y axis shows the relative activity (%) of the optimal temperature. **C**: Effect of pH on the activity of Hl-Amy. The X axis represents the value of pH and the Y axis shows the relative activity (%) of the optimal pH.

To characterize the activity of Hl-Amy on hydrolysis of glycosides in starch, a unit of enzyme activity is defined as the enzyme amount required to produce 1 μmol glucose per minute at the optimal temperature and pH. The absorbance of Hl-Amy at A_562_ was 0.836, which was substituted into the standard curve y = 1.966x+0.2246 (R^2^ = 0.997) (Fig 6A) to obtain x = 0.56 mg/mL; thus, the activity of amylase *Hl-Amy* was calculated to be 62.2 U/mg. By further analysis, the optimal temperatures and optimal pH for Hl-Amy were found to be 55°C (Fig 6B) and 6.0 (Fig 6C), respectively. In addition, Hl-Amy showed stronger activity in acidic conditions than in neutral and alkaline conditions.

## Discussion

In this study, the α-amylase (*Hl-Amy*) gene from tropical sea cucumber (*H*. *leucospilota*) was identified followed by characterization of its structural and phylogenetical features, expressional profiles and biological function. To the best of our knowledge, this study is the first to perform cDNA cloning and functional analysis to describe a full-length cDNA of amylase obtained in echinoderms. According to the prediction of the SMART program, an Aamy feature and an Aamy_C feature are presented in the a.a. sequence of Hl-Amy (Fig 1B). In general, these enzymes are composed of three domains, namely, domain A with a TIM barrel containing the active site residues and chloride ion-binding site [22], domain B with a long loop region inserted between the third β-strand and the α-helix of domain A that contains calcium-binding sites, and domain C with a C-terminal β-sheet domain that appears to show some variability in sequence and length between amylases (Figs 1A and 2C) [23]. The predicted SMART Aamy feature include the domain A and B, while SMART Aamy_C feature represents the domain C (Fig 1B). Although all α-amylases possess the same catalytic function, they can vary with respect to sequence. Multiple sequence alignment of amylases showed that Asp^210^, Glu^246^ and Asp^312^ within domain A were found in the α-amylases from human (*H*. *sapiens*), Norway rat (*Rattus norvegicus*), zebrafish (*Danio rerio*), purple sea urchin (*S*. *purpuratus*), common starfish (*A*. *ruben*) and *H*. *leucospilota* (Fig 2A), indicating that these conserved a.a are the main catalytic activity sites of this enzyme.

The copy number of amylase gene is different between populations of high- and low-starch diets with positive correlation [24]. In addition, the transcript expression of amylase is reported to be influenced by the amounts of carbohydrate in feeding diet [25]. After fertilization, the sea cucumber embryos mainly develop following the stages of blastula, gastrula, auricularia, doliolaria and pentactula and finally become juveniles [17]. The functional gut of *H*. *leucospilota* appeared at the developmental stage of the auricularia 36 h after fertilization, when the larva were fed a mixture of 1–30,000 cells/ml *Chaetoceros muelleri* and 0.5–1.0 g/m^3^ *Saccharomyces cerevisiae* twice a day[16]. During embryonic and larval development, the highest mRNA expression of *Hl-Amy* was observed at the blastula stage, then it was decreased gradually from the early gastrula to the mid auricularia stages and reached the lowest level at the doliolaria stage; after that stage, it was increased again. The ontogeny of the *Hl-Amy* transcripts is similar to that of *H*. *leucospilota* trehalase [17] and was consistent with those in numerous fishes, which is decreasing throughout the larval developmental stages to stay at low levels and has been considered an indicator of digestive maturity [18]. In many studies, the amylase mRNA expressions were shown decrease with the embryonic development concurrently with those of amylase activities [18, 26–28]. This phenomenon is very different from other digestive enzymes (e.g. trypsin, lipase and phospholipase), which were increased until the end of the larva period [29]. Thus, it is speculated that the beginning of amylase mRNA or amylase activity rising again indicates the digestive maturity. In our study, the amylase mRNA expression reached a lowest level at the developmental stage of doliolaria and started to increase at pentactula, indicating that digestive maturity of the sea cucumber appeared at the developmental stage of pentactula, which can help to understand the weaning process and an effective feeding strategy. Interestingly, food resources and species were increased, but the mRNA expression of amylase was decreased throughout larval development. In teleost fishes, such as seabream (*Sparus aurata*), turbot (*Scophthalmus maximus*) and redfish (*Sebastes mentella*), and four prickleback species [30–32], it has been proven that α-amylase is genetically programmed in the first days before hatching for utilizing the glycogen in the yolk-sac, providing a possible mechanism for the high activity and expression of α-amylase in the embryo and larvae before they began to feed. It is speculated that some disaccharides and monosaccharides in the larval food can be directly hydrolyzed into glucose for absorption and utilization, limiting the use of α-amylase in these stages. A previous study in Indianmeal moth (*Plodia interpunctella*) showed that the food with high levels of glucose may reduce the postembryonic α-amylase activities [33]. In contrast, the ruminants fed the high energy/high starch diet tended to have more pancreatic α-amylase protein and had greater protein activity but lower α-amylase mRNA levels [34].

By tissue distribution analysis, the highest expression level of *Hl-Amy* mRNA was observed in the intestine followed by the transverse vessel (Fig 3A). Intestine is the main apparatus for food digestion and absorption in sea cucumber, and transverse vessels are hemal vessels along the gut, sinus and lacunae [35]. However, studies regarding the function of transverse vessels are still limited. Based on its high expression of digestive enzymes, such as amylase and trehalase [17], the transverse vessel is speculated to be one of the apparatuses in nutrient absorption and transportation. In addition, the *IS*H result show that the *Hl-Amy* mRNA positive signal were mainly appeared in the foregut and midgut, indicating that carbohydrate from starchy foods were primarily digested and absorbed in the foregut and midgut of *H*. *leucospilota*.

To investigate the biological activities of amylase in *H*. *leucospilota*, recombinant Hl-Amy protein was expressed as a C-terminal His-tagged fusion protein in *E*. *coli* using codon optimization. To the best of our knowledge, this report describes the first successful expression of an α-amylase from marine animals in the prokaryotic expression system. Although the functions and activities of α-amylase from marine animals have been investigated in a variety of species [36–39], the prokaryotic *E*. *coli* system has not yet been applied for generation of recombinant α-amylase protein, despite it is considered as a high-efficient method for heterogenous protein expression and have been applied for multiple sea cucumber protein expression [40–43]. However, the Hl-Amy protein can only be successfully expressed in the *E*. *coli* system after nucleotide codon optimization but not with its original sequence. It is possibly due to the codon usage bias is different between bacterial and marine animals.

After the activity assay, the optimal pH and temperature for purified amylase *Hl-Amy* were found to be 6.0 and 55°C, respectively, with an activity of 62.2 U/mg. The optimal pH of amylases is species-specific, with a wide range of 3.5–7.5. The optimal pH of Hl-Amy (6.0) is similar to those amylases from copepod (*Acartia clause*) (6.0) [44], short-necked clam (*Ruditapes philippinarum*) (6.2) [37] and small abalone (*Sulculus diversicolor*) (6.0) [36]. The optimal pH of α-amylase is 6.9 in those from the human sliver and pancreas, and the dog pancreas in mammals [45], 7.5 in those from the ostrich (*Struthio camelus*) and chicken (*Gallus gallus*) pancreas in birds [46, 47], 5.2–5.4 in that from beetle (*Callosbruchus chinensis*) in insect [48], 7.3 in those from crabs (*Uca minax*, *Uca pugnax*, and *Uca pugilator*) in crustacean [49], and 3.5 in that from Gram-positive bacteria (*Bacillus acidocaldarius*) [50]. The optimal temperatures of amylases are also species-specific, but most of these temperatures are between 35 to 45°C. The optimal temperature for the recombinant Hl-Amy (55°C) obtained in this study, which is similar to those of small abalone (*S*. *diversicolor*) amylase-AII (50°C) [36], higher than those α-amylases from copepod (*A*. *clause*, 40°C) [44], short-necked clam (*R*. *philippinarum*, 36°C) [37], Asian green mussel (*Perna viridis*, 43°C) [38], beetle (*C*. *chinensis*, 36–40°C) [48] and shrimp (*Penaeus japonicas*, 40°C) [39] but lower than those from Gram-positive bacteria (*Bacillus*. *lentus*, 70°C [51], *Bacillus*. *licheniformis*, 76°C [52] and *B*. *acidocaldarius*, 75°C [50]).

In a human study, it is well-known that the concentration of α-amylase in different body fluids, such as blood, saliva or urine, is the typical indicator of a number of common diseases [53]. The rise or fall of the enzyme concentrations or activities indicate the onset of pancreatitis, pancreatic cancer, mumps, stress or depression, toxemia in pregnancy, and liver cirrhosis [54–57]. According to this trait of α-amylase, a biosensor to detect the above mentioned disease was developed [58]. In the Pacific oyster (*Crassostrea gigas*), it has been proven that the disruption of amylase gene expression can lead to delayed gametogenesis with an associated reduction in reproductive effort [59]. To date, no effective method has been found to identify the sex and sexual maturity of sea cucumbers without damaging them. Therefore, whether sea cucumber with mature gonads can be detected by α-amylase biosensors warrants further research.

## Conclusions

In conclusion, the first echinoderm α-amylase was identified from the tropical sea cucumber *H*. *leucospilota*. The full-length cDNA sequence of *Hl-Amy* was obtained, and the structural and phylogenetic features of Hl-Amy were further analyzed. We detected the tissue distribution and cellular expression profiles of *Hl-Amy* mRNA. *Hl-Amy* mRNA expression was further measured during embryonic and larval development. Recombinant Hl-Amy protein was expressed in *E*. *coli* by codon optimization, and this report is the first to describe the expression of an α-amylase from marine animals in the prokaryotic expression system. After purification and activity assays, the optimal pH and temperature for recombinant Hl-Amy were found to be 6.0 and 55°C, respectively, with an activity of 62.2 U/mg. Overall, the results of our study help to elucidate the characteristics and function of α-amylase in a tropical sea cucumber, which can provide a better understanding of the digestive system of sea cucumber and help to optimize heterogenous prokaryotic systems for producing recombinant enzymes from marine animals.

## Supporting information

S1 Table Primer sequence information. (DOCX)

S1 Data The 1494 bp Hl-Amy unigenes in the previously constructed H. leucospilota transcriptome. (DOCX)

S2 Data The original and optimized nucleotide sequences of Hl-Amy. (DOCX)

S1 Raw images (PDF)
