# Supplementary material for: First echinoderm alpha-amylase from a tropical sea cucumber (Holothuria leucospilota): Molecular cloning, tissue distribution, cellular localization and functional production in a heterogenous E.coli system with codon optimization
Source: PLoS One. 2020 Sep 15;15(9):e0239044. doi: 10.1371/journal.pone.0239044 (PMC7491741; doi:10.1371/journal.pone.0239044)
Supplement: S1 Table — (DOCX) [file pone.0239044.s001.docx]

| Primers | Primer sequences (5’-3’) |
| --- | --- |
| *For cDNA cloning* |  |
| **Amy-M-F** | TCCAAGAAAGCAACCGACA |
| **Amy-M-R** | GAGCCTTCAACAGACTGAAAAT |
| **A-3’RACE-F1** | ACGATGGACCTCCGTCTAAT |
| **A-3’RACE-F2** | ATGGAAATCAACAAGTGGCA |
| **A-3’RACE-F3** | CCGACTTCACCATTGCTTT |
| **A-3’RACE-F4** | TTCCTCCTGTCACTTTCTTTCA |
| *For qPCR* |  |
| **q-Amy-F** | ATGGAAATCAACAAGTGGCA |
| **q-Amy-R** | TCGGCGTAACCTTCAGAGT |
| **qβ-actin-F** | CCAGAGGAACACCCAGTC |
| **qβ-actin-R** | AGGGCGTAACCTTCATAG |
| *For ISH probe* |  |
| **p-Amy-F** | GGAATACAAAATGGCTAACGC |
| **p-Amy-R** | AAAGCAATGGTGAAGTCGG |
| *For codon optimization* |  |
| **Amy-CO-F1** | TGGAAATGGACAGACGTAGCTCTT |
| **Amy-CO-F2** | CAGTTTGATACCAACGCGGTGGGCGATCGCGAAACCATTGTGCAGCTGTTTAGCTGGAAATGGACAGACGTAGCTCTT |
| **Amy-CO-F3** | CGCGGATCCGAATTCGAGCTCCAGTTTGATACCAACGCGGTGGG |
| **Amy-Xho1-R** | GTGGTGGTGGTGGTGCTCGAGTCTATATGAAAGAAAGTGAC |

Table 1. Primer sequences information.
