# Supplementary material for: First echinoderm alpha-amylase from a tropical sea cucumber (Holothuria leucospilota): Molecular cloning, tissue distribution, cellular localization and functional production in a heterogenous E.coli system with codon optimization
Source: PLoS One. 2020 Sep 15;15(9):e0239044. doi: 10.1371/journal.pone.0239044 (PMC7491741; doi:10.1371/journal.pone.0239044)
Supplement: S1 Data — (DOCX) [file pone.0239044.s002.docx]

S1 Data. The 1494 bp *Hl-Amy* unigenes in the previously constructed H. leucospilota transcriptome.

Amylase

Unigene34210_All

CDS

ATGCAGACATTTATGATACTCTTCATCCTTGGGGTGGCCAATGGCCAGTTCGATACCAACGCCGTTGGAGATCGTGAAACAATAGTGCAGCTTTTCAGTTGGAAATGGACAGACGTAGCTCTTGAATGTGAAAGATTCTTGGGGCCCAACGGATATGGAGGGGTACAAGTATCACCACCAAACGACCACACTATCATGAATGATCCATTTCGACCGTGGTGGGAGAGATATCAAGTCGCAGGGTACAACCTCGTAAGTCGCAGTGGTGACGAAAACGAGTTTGCGGACATGGTGGAGAGATGTAATCAAGCCAACGTTAGAATATATGTAGACGCTGTCATTAACCACATGGCGTTCTTTGGTGGGGATTCAGCAAGCGGGGAGCCTTTTAATCCGGACGAACTAGATTATCCAACTGTGCCATACACGGAGGAAGATTTCAGCGTTTATTACGGTCTCTGTAACACCACGAACCAAGATATTCTCAACCAGTCGAGTGTTAAGGAGTTACGCGACTGTAACCTGCTGGCCCTTAAGGACCTCGCCCAACATGAGGAGAGGGTGAGATCAAAGGTAGCAGCCTACTTGAACAAGATGATTGATATCGGAGTTGCCGGCTTCCGTCTTGATGCTGCCAAACACATGTGGCCGGATGATTTAGAAAACATCTACGGACGACTGAATGAGTTGAAAGCGGACCACTTTGAAGAGGGGTCTAAAGCATTGCTCTACCACGAGGTCATTGATAAAGGTCAGGACCCGATAAGAGCTACGGAGTACACACATCTAGGAAGGGTAACCGAATTCAACTACGGACCATTGATAGTTGATTGTATACGTAGGCACACTCCCTTGAAAGATTTCGGGAGGTTTAATTTCGCCGAGTCTTGGGAGCTCCTTCCCAGTGGCGAAGCTGTAAGTTTTATTGACAACCATGATAATCAGAGAGGAGAAGGCCAAGAAGAGATTGTGAATTTTAAAGAACCCAAGGAATACAAAATGGCTAACGCTCTCATGCTGGCGTGGCCTTACGGTATCAACAGGGTCATGTCAAGTTATGAATTTGAGACATCTGACGATGGACCTCCGTCTAATGAAGACGGCGATCTTCTGTCGCCCGAAATAGATGAAGACGGCTTATGTACCGGAGGTTGGGTATGCGAACACCGATGGAGGGTGATCAAAAATATGGTCAAGTACCAGAACGTGGTGCGTAAAGAATCTGTTATGAATTGGTGGGACAATGGAAATCAACAAGTGGCATTCGGTCGAGGCAAAAAGGGGTTTTTTGTAATGAATAATGAACTCGAACAGAATCTGACCGAAACCATAATGACAGGCCTACCACAGGGTGAATACTGCAATGTGATACTAGGTGAAATGACCGATGGCGAGTGTTCGGGACCAACTGTACAAGTCAACTCTGAAGGTTACGCCGACTTCACCATTGCTTTTGATTCCGAAGAACCTATGGTTGCCATTCATGTTGATGCC
