# Supplementary material for: First echinoderm alpha-amylase from a tropical sea cucumber (Holothuria leucospilota): Molecular cloning, tissue distribution, cellular localization and functional production in a heterogenous E.coli system with codon optimization
Source: PLoS One. 2020 Sep 15;15(9):e0239044. doi: 10.1371/journal.pone.0239044 (PMC7491741; doi:10.1371/journal.pone.0239044)
Supplement: S2 Data — (DOCX) [file pone.0239044.s003.docx]

S2 Data. The original and optimized nucleotide sequences of *Hl-Amy*.

*Hl-Amy* original sequence

   1 CAGTTCGATA CCAACGCCGT TGGAGATCGT GAAACAATAG TGCAGCTTTT CAGTTGGAAA

61 TGGACAGACG TAGCTCTTGA ATGTGAAAGA TTCTTGGGGC CCAACGGATA TGGAGGGGTA

121 CAAGTATCAC CACCAAACGA CCACACTATC ATGAATGATC CATTTCGACC GTGGTGGGAG

181 AGATATCAAG TCGCAGGGTA CAACCTCGTA AGTCGCAGTG GTGACGAAAA CGAGTTTGCG

241 GACATGGTGG AGAGATGTAA TCAAGCCAAC GTTAGAATAT ATGTAGACGC CGTCATTAAC

301 CACATGGCGT TCTTTGGTGG GGATTCAGCA AGCGGGGAGC CTTTTAATCC GGACGAACTA

361 GATTATCCAA CTGTGCCATA CACGGAGGAA GATTTCAGCG TTTATTACGG TCTCTGTAGC

421 ACCACGAACC AAGATATTCT CAACCAGTCG AGTGTTAAGG AGTTACGCGA CTGTAACCTG

481 CTGGCCCTTA AGGACCTCGC CCAACATGAG GAGAGGGTGA GATCAAAGGT AGCAGCCTAC

541 TTGAACAAGA TGATTGATAT CGGAGTTGCC GGCTTCCGTC TTGATGCTGC CAAACACATG

601 TGGCCGGATG ATTTAGAAAA CATCTACGGA CGACTGAATG AGTTGAAAGC GGACCACTTT

661 GAAGAGGGGT CTAAAGCATT GCTCTACCAC GAGGTCATTG ATAAAGGTCA GGACCCGATA

721 AGAGCTACGG AGTACACACA TCTAGGAAGG GTAACCGAAT TCAACTACGG ACCATTGATA

781 GTTGATTGTA TACGTAGGCA CACTCCCTTG AAAGATTTCG GGAGGTTTAA TTTCGCCGAG

841 TCTTGGGAGC TCCTTCCCAG TGGCGAAGCT GTAAGTTTTA TTGACAACCA TGATAATCAG

901 AGAGGAGAAG GCCAAGAAGA GATTGTGAAT TTTAAAGAAC CCAAGGAATA CAAAATGGCT

961 AACGCTCTCA TGCTGGCGTG GCCTTACGGT ATCAACAGGG TCATGTCAAG TTATGAATTT

1021 GAGACATCTG ACGATGGACC TCCGTCTAAT GAAGACGGCG ATCTTCTGTC GCCCGAAATA

1081 GATGAAGACG GCTTATGTAC CGGAGGTTGG GTATGCGAAC ACCGATGGAG GGTGATCAAA

1141 AATATGGTCA AGTACCAGAA CGTGGTGCGC AAAGAATCTG TTATGAATTG GTGGGACAAT

1201 GGAAATCAAC AAGTGGCATT CGGTCGAGGC AAAAAGGGGT TTTTTGTAAT GAATAATGAA

1261 CTCGAACAGA ATCTGACCGA GACCATAATG ACAGGCCTAC CACAGGGTGA ATACTGTAAT

1321 GTAATATTAG GTGAAATGAC CGATGGCGAG TGTTCGGGAC CAACTGTACA AGTCAACTCT

1381 GAAGGTTACG CCGACTTCAC CATTGCTTTT GATTCCGAAG AACCTATGGT TGCCATCCAT

1441 GTTGATGCCC TGGTCGCTGG TACGGGAAAC GTACATGTTG CTTCCTTTGT GGTAATACTG

1501 TTGGCGTTCC TCCTGTCACC TTCTTTCATA TAG

*Hl-Amy* optimized sequence

1 CAGTT**T**GATA CCAACGC**G**GT **G**GG**C**GATCG**C** GAAAC**C**AT**T**G TGCAGCT**G**TT **T**AG**C**TGGAAA 
      61 TGGACAGACG TAGCTCTTGA ATGTGAAAGA TTCTTGGGGC CCAACGGATA TGGAGGGGTA 
     121 CAAGTATCAC CACCAAACGA CCACACTATC ATGAATGATC CATTTCGACC GTGGTGGGAG 
     181 AGATATCAAG TCGCAGGGTA CAACCTCGTA AGTCGCAGTG GTGACGAAAA CGAGTTTGCG 
     241 GACATGGTGG AGAGATGTAA TCAAGCCAAC GTTAGAATAT ATGTAGACGC CGTCATTAAC 
     301 CACATGGCGT TCTTTGGTGG GGATTCAGCA AGCGGGGAGC CTTTTAATCC GGACGAACTA 
     361 GATTATCCAA CTGTGCCATA CACGGAGGAA GATTTCAGCG TTTATTACGG TCTCTGTAGC 
     421 ACCACGAACC AAGATATTCT CAACCAGTCG AGTGTTAAGG AGTTACGCGA CTGTAACCTG 
     481 CTGGCCCTTA AGGACCTCGC CCAACATGAG GAGAGGGTGA GATCAAAGGT AGCAGCCTAC 
     541 TTGAACAAGA TGATTGATAT CGGAGTTGCC GGCTTCCGTC TTGATGCTGC CAAACACATG 
     601 TGGCCGGATG ATTTAGAAAA CATCTACGGA CGACTGAATG AGTTGAAAGC GGACCACTTT 
     661 GAAGAGGGGT CTAAAGCATT GCTCTACCAC GAGGTCATTG ATAAAGGTCA GGACCCGATA 
     721 AGAGCTACGG AGTACACACA TCTAGGAAGG GTAACCGAAT TCAACTACGG ACCATTGATA 
     781 GTTGATTGTA TACGTAGGCA CACTCCCTTG AAAGATTTCG GGAGGTTTAA TTTCGCCGAG 
     841 TCTTGGGAGC TCCTTCCCAG TGGCGAAGCT GTAAGTTTTA TTGACAACCA TGATAATCAG 
     901 AGAGGAGAAG GCCAAGAAGA GATTGTGAAT TTTAAAGAAC CCAAGGAATA CAAAATGGCT 
     961 AACGCTCTCA TGCTGGCGTG GCCTTACGGT ATCAACAGGG TCATGTCAAG TTATGAATTT 
    1021 GAGACATCTG ACGATGGACC TCCGTCTAAT GAAGACGGCG ATCTTCTGTC GCCCGAAATA 
    1081 GATGAAGACG GCTTATGTAC CGGAGGTTGG GTATGCGAAC ACCGATGGAG GGTGATCAAA 
    1141 AATATGGTCA AGTACCAGAA CGTGGTGCGC AAAGAATCTG TTATGAATTG GTGGGACAAT 
    1201 GGAAATCAAC AAGTGGCATT CGGTCGAGGC AAAAAGGGGT TTTTTGTAAT GAATAATGAA 
    1261 CTCGAACAGA ATCTGACCGA GACCATAATG ACAGGCCTAC CACAGGGTGA ATACTGTAAT 
    1321 GTAATATTAG GTGAAATGAC CGATGGCGAG TGTTCGGGAC CAACTGTACA AGTCAACTCT 
    1381 GAAGGTTACG CCGACTTCAC CATTGCTTTT GATTCCGAAG AACCTATGGT TGCCATCCAT 
    1441 GTTGATGCCC TGGTCGCTGG TACGGGAAAC GTACATGTTG CTTCCTTTGT GGTAATACTG 
    1501 TTGGCGTTCC TCCTGTCACC TTCTTTCATA TAG
