# Supplementary figures and images for: First echinoderm alpha-amylase from a tropical sea cucumber (Holothuria leucospilota): Molecular cloning, tissue distribution, cellular localization and functional production in a heterogenous E.coli system with codon optimization
Source: PLoS One. 2020 Sep 15;15(9):e0239044. doi: 10.1371/journal.pone.0239044 (PMC7491741; doi:10.1371/journal.pone.0239044)

Raw-figure 5

A

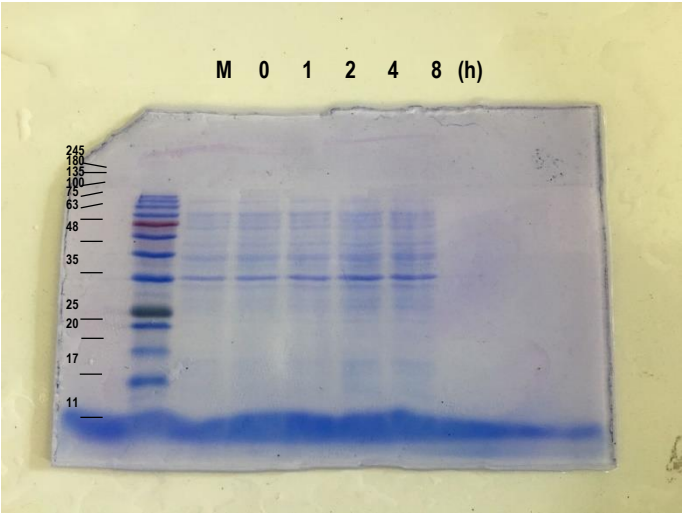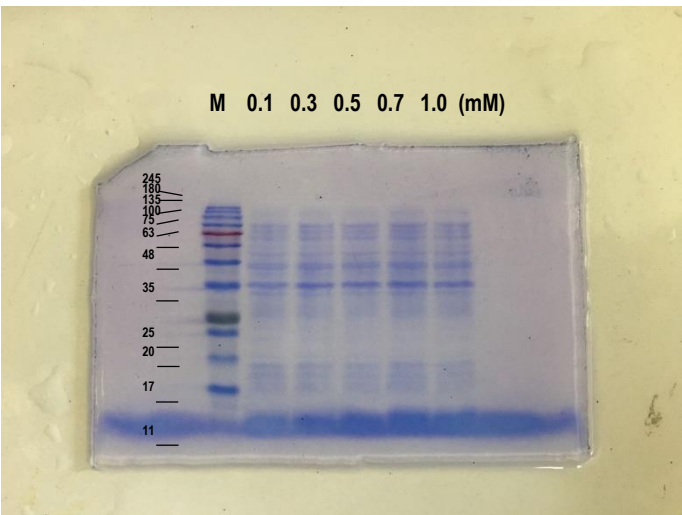

C

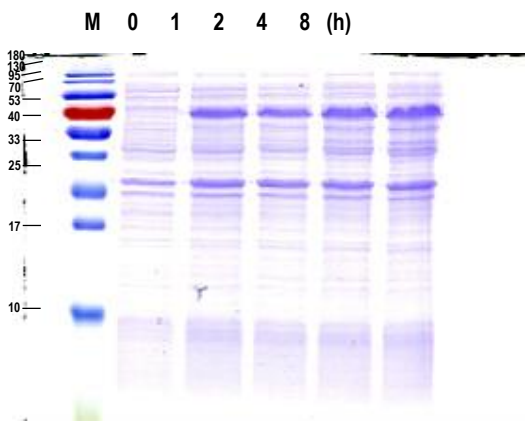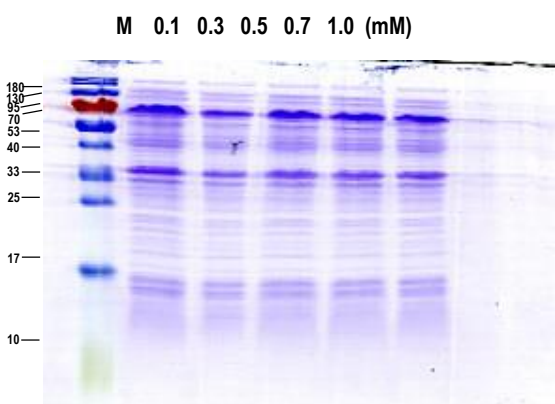

D

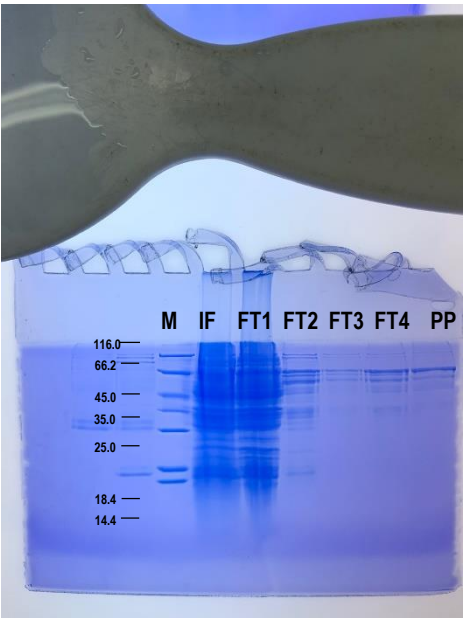

Supplement: S1 Raw images — (PDF) [file pone.0239044.s004.pdf]
